# The Crystal Structure of the *Escherichia coli* Autoinducer-2 Processing Protein LsrF

**DOI:** 10.1371/journal.pone.0006820

**Published:** 2009-08-28

**Authors:** Zamia Diaz, Karina B. Xavier, Stephen T. Miller

**Affiliations:** 1 Department of Chemistry and Biochemistry, Swarthmore College, Swarthmore, Pennsylvania, United States of America; 2 Instituto Gulbenkian de Ciência, Oeiras, Portugal; 3 Instituto de Tecnologia Química e Biológica, Estação Agronómica Nacional, Oeiras, Portugal; Duke University Medical Center, United States of America

## Abstract

Many bacteria produce and respond to the quorum sensing signal autoinducer-2 (AI-2). *Escherichia coli* and *Salmonella typhimurium* are among the species with the *lsr* operon, an operon containing AI-2 transport and processing genes that are up regulated in response to AI-2. One of the Lsr proteins, LsrF, has been implicated in processing the phosphorylated form of AI-2. Here, we present the structure of LsrF, unliganded and in complex with two phospho-AI-2 analogues, ribose-5-phosphate and ribulose-5-phosphate. The crystal structure shows that LsrF is a decamer of (αβ)_8_-barrels that exhibit a previously unseen N-terminal domain swap and have high structural homology with aldolases that process phosphorylated sugars. Ligand binding sites and key catalytic residues are structurally conserved, strongly implicating LsrF as a class I aldolase.

## Introduction

Many bacterial species control expression of specific genes thorough the production, release, and detection of small signal molecules called autoinducers. This process, termed quorum sensing, allows bacteria to regulate behavior in a population-dependant manner, effectively coordinating their activity. Behaviors regulated by quorum sensing include bioluminescence, biofilm formation, and production of virulence factors [1].

While autoinducer production and recognition is generally species specific, autoinducer-2 (AI-2) has been shown to be produced and recognized by a variety of bacterial species, both Gram-positive and Gram-negative. First identified as a regulator of bioluminescence in *Vibrio harveyi* [2], [3], AI-2 has been shown to control a wide variety of behaviors in different species, including motility in *Helicobacter pylori* [4], division, stress response, and biofilm formation in *Streptococcus mutans* [5], [6], virulence and formation of biofilms in *Vibrio cholerae* [7]–[9] and *Staphylococcus epidermis* [10], social and pluricellular behavior of *Bacillus subtilis* [11], and virulence in *Erwinia carotovora* ssp. *carotovora* [12]. Since many species produce and respond to AI-2, it is believed to facilitate interspecies communication, potentially allowing bacteria to modulate gene expression in response to both the concentration and species composition of bacteria in the local environment; indeed, some species of bacteria have been shown to respond to AI-2 produced by other species in co-culture experiments [13], [14].

AI-2 is produced by the highly conserved synthase LuxS, which catalyzes the production of 4,5-dihydroxy-2,3-pentanedione (DPD) from *S*-ribosylhomocysteine [2]. Crystal structures of AI-2 receptor/ligand complexes from *V. harveyi* [15], *S. typhimurium* [16], and *S. meliloti* [13] have shown that these species recognize chemically distinct DPD adducts as AI-2: (2*S*, 4*S*)-2-methyl-2,3,3,4-tetrahydroxyterahydrofuran-borate in the case of *V. harveyi* and (2*R*, 4*S*)-2-methyl-2,3,3,4-tetrahydroxyterahydrofuran in the case of *S. typhimurium* and *S. meliloti*. The known forms of AI-2 are able to interconvert spontaneously in solution, suggesting that a mix of DPD-derived molecules exists in environments with LuxS-containing bacteria [16]; however, because the different forms of AI-2 can interconvert, bacteria that recognize chemically distinct forms of AI-2 can nonetheless communicate with each other [14].

**Figure 1:**
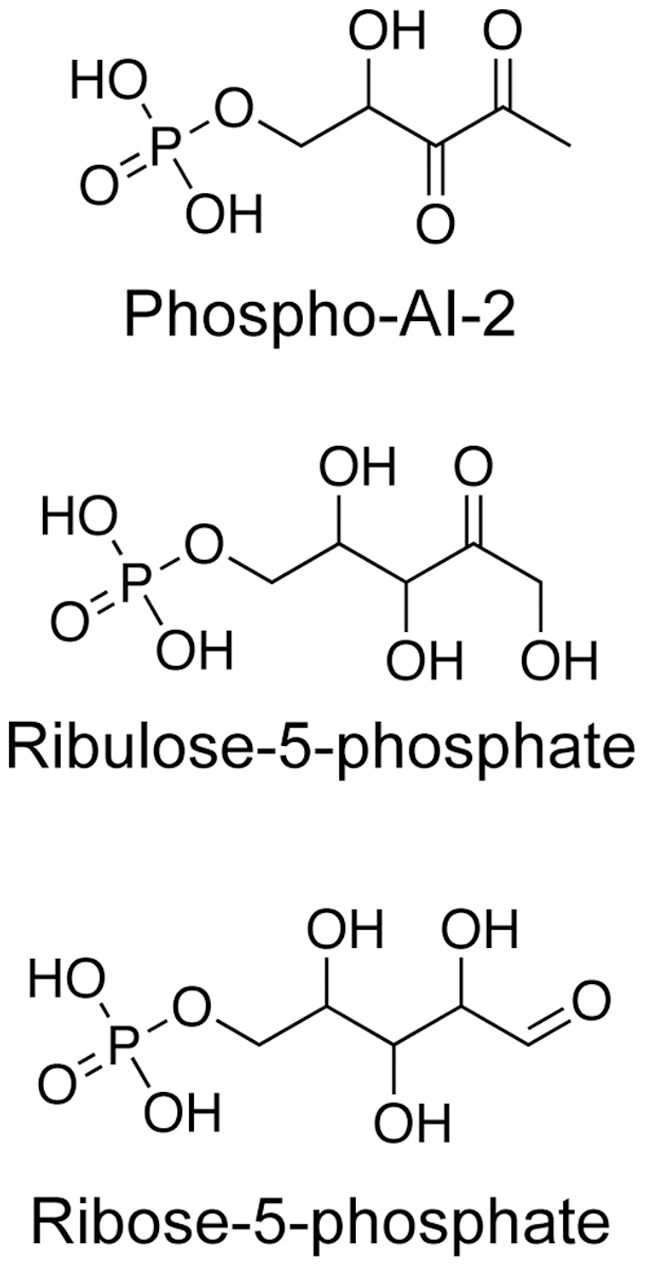
Structures of phospho-AI-2 and two analogues, ribulose-5-phosphate and ribose-5-phosphate.

While AI-2 has been shown to act as a signaling molecule in many bacterial species [17], [18], the molecular details of AI-2 recognition and response have been studied in only a small number of species including *E. coli* [19], [20], *S. typhimurium* [21], [22], *Sinorhizobium meliloti* [13], *V. cholerae* [9], [23]–[25], and *V. harveyi* [3], [15], [26], [27]. *E. coli* and *S. typhimurium* share an operon, named *lsr* (for LuxS Regulated), that consists of *lsrA*, *lsrB*, *LsrC*, *lsrD*, *lsrF*, and *lsr*G (and, in the case of *S. typhimurium*, *lsrE*) and is responsible for the recognition and transport of AI-2. (Two additional genes involved in regulation of the *lsr* operon, *lsrR* and *lsrK*, are adjacent but are transcribed divergently.) These species internalize AI-2 via an ABC transporter complex comprised of LsrA, LsrB, LsrC, and LsrD [19], [22]. Once internalized, AI-2 is phosphorylated at the C5 position by the kinase LsrK, giving rise to phospho-AI-2 (P-AI-2, Fig. 1) [20], [21]. It is this phosphorylated form of AI-2 that binds to the repressor LsrR, inactivating repression and increasing transcription of the *lsr* operon; thus, the operon acts as a positive feedback loop, importing more AI-2 in response to detection of P-AI-2 [20]. Two additional genes in the *lsr* operon, *lsrF* and *lsrG*, are present in both *E. coli and S. typhimurium* and have been implicated in AI-2 processing while a final gene, *lsrE*, is found in the *S. typhimurium lsr* operon but not in *E. coli* [21]. *lsrE* has homology to epimerases, but deleting *lsrE* in *S. typhimurium* has no detectable impact on AI-2 uptake or transcription of the *lsr* operon.

Previous genetic studies demonstrated that the LsrF and LsrG proteins are involved in terminating the AI-2-depent induction of the *lsr* operon. Mutants lacking LsrF or LsrG show increased transcription of the *lsr* operon, suggesting that these proteins process P-AI-2, thus reducing the concentration of P-AI-2 in the cell and restoring the repressor function of *lsrR*. Importantly, the increase in *lsr* transcription observed in the absence of LsrF or LsrG is AI-2 dependent, and over-production of LsrF or LsrG decreases the transcription of the operon to levels lower than in the wild type. The suggestion that LsrF plays a role in P-AI-2 processing is further supported by sequence homology with aldolase enzymes that process phosphorylated sugars [21]. Subsequent studies have shown that LsrG does, in fact, catalyze a reaction with P-AI-2 as a substrate, yielding 2-phosphoglycolic acid and an additional, as yet unidentified three-carbon fragment, raising the possibility that LsrF does not act directly upon P-AI-2 but rather a product of the LsrG reaction or another P-AI-2 adduct [20].

Aldolases catalyze the formation or cleavage of carbon-carbon bonds and are classified in two families based on their mechanism [28]. Class I aldolases act through the formation of a Schiff base with the substrate, while class II aldolases require metal co-factors. The best studied of the class I aldolases is fructose-1,6-bisphosphate aldolase (FBPA), which catalyzes the cleavage of fructose-1,6-bisphosphate into glyceraldehyde-3-phosphate and dihydroxyacetone phosphate in glycolysis. Crystal structures have been determined for FBPA [29], [30] and a variety of other class I aldolases including 2-amino-3,7-dideoxy-D-*threo*-hept-6-ulosonic acid (ADH) synthase, which catalyzes a transaldol reaction of 6-deoxy-5-ketofructose-1-phosphate with L-aspartate semialdehyde to yield ADH [31], and D-2-deoxyribose-5-phosphate aldolase (DERA), which catalyzes the reversible aldol reaction between acetaldehyde and D-glyceraldehyde-3-phosphate to generate D-2-dexoyribose-5-phosphate [32]. These structures reveal that the class I aldolases share a common fold, classified as a TIM α/β-barrel in SCOP [33], and a structurally conserved catalytic lysine responsible for Schiff base formation.

While sequence analysis suggests that LsrF will function as a class I aldolase and genetic data suggests LsrF is involved in P-AI-2 processing, the details of the role LsrF plays in processing P-AI-2 are not known [21]. To begin addressing this question, we have determined the crystal structure of LsrF, alone and in complex with the P-AI-2 analogues ribose-5-phosphate and ribulose-5-phosphate (Fig. 1). The structure reveals a decameric complex of TIM α/β-barrels. Despite strong structural homology to FBPA from *Thermoproteus tenax* and ADH synthase from *Methanocaldococcus jannaschii*, the subunits participate in a form of domain swapping previously unseen in aldolase complexes. Key catalytic residues in these class I aldolases are structurally conserved in LsrF, and both P-AI-2 analogues bind LsrF in the canonical aldolase active site, strongly implicating LsrF as a class I aldolase.

## Results

### LsrF Structure

**Figure 2:**
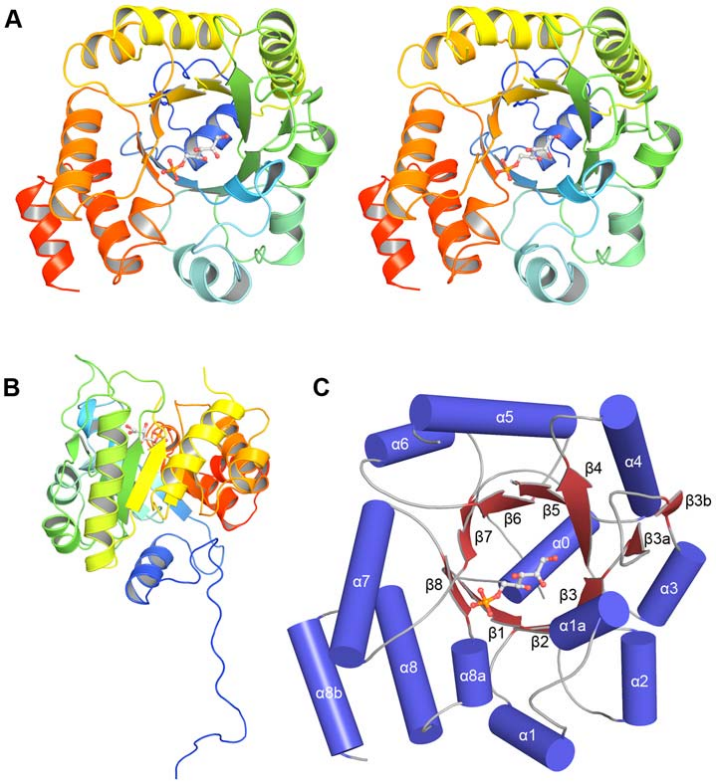
Structure of a single LsrF chain. A. Stereoview of a single (α/β)8-barrel subunit with protein backbone in cartoon representation and bound P-AI-2 analogue (ribulose-5-phosphate) as ball-and-stick. The protein backbone is rainbow colored, with blue at the N-terminus and red at the C-terminus. B. Rotated view of the subunit (approximately 90°) highlighting the N-terminal residues that extend away from the (αβ)8-barrel and are swapped with the adjacent 2-fold related subunit. C. Identification of the components of the (αβ)8-barrel, with α-helices as blue cylinders and β-sheets as red arrows. The bound ligand (ribulose-5-phosphate) is shown in ball-and-stick format.

**Figure 3:**
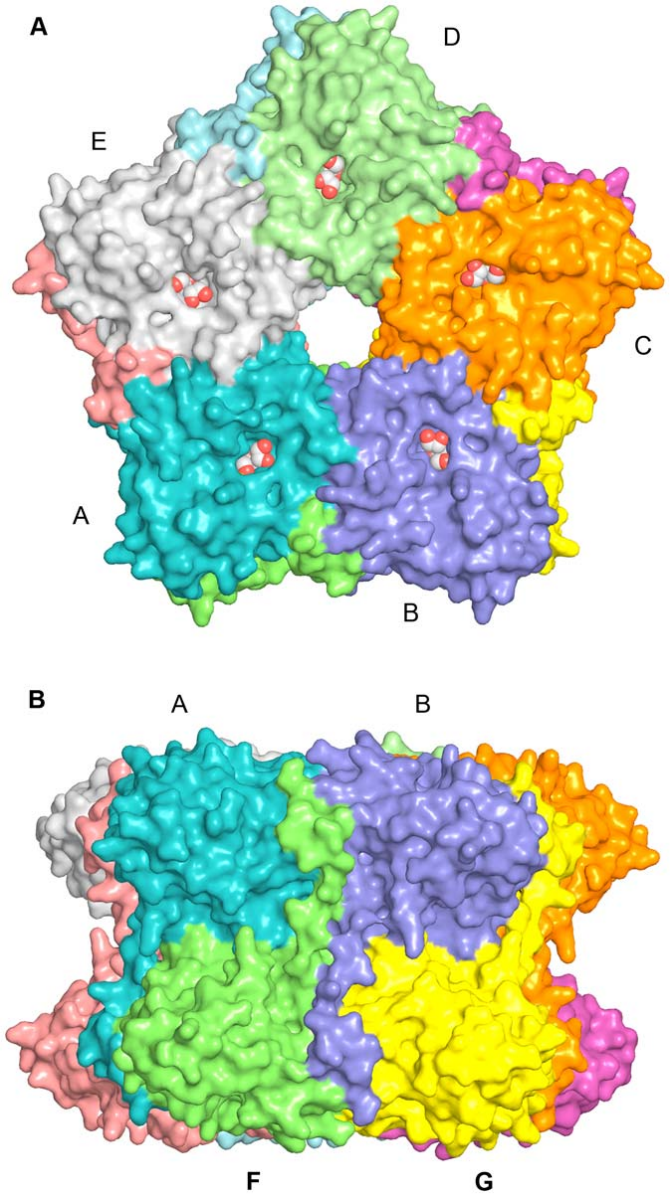
Structure of the LsrF decamer. A. Surface representation of the LsrF decamer, viewed down the 5-fold symmetry axis, with each monomer a different color. The bound ligand (ribose-5-phosphate) is visible in the center of the (αβ)_8_-barrel, and is shown in ball-and-stick format. B. Perpendicular view of the LsrF decamer along a two-fold axis.

**Figure 4:**
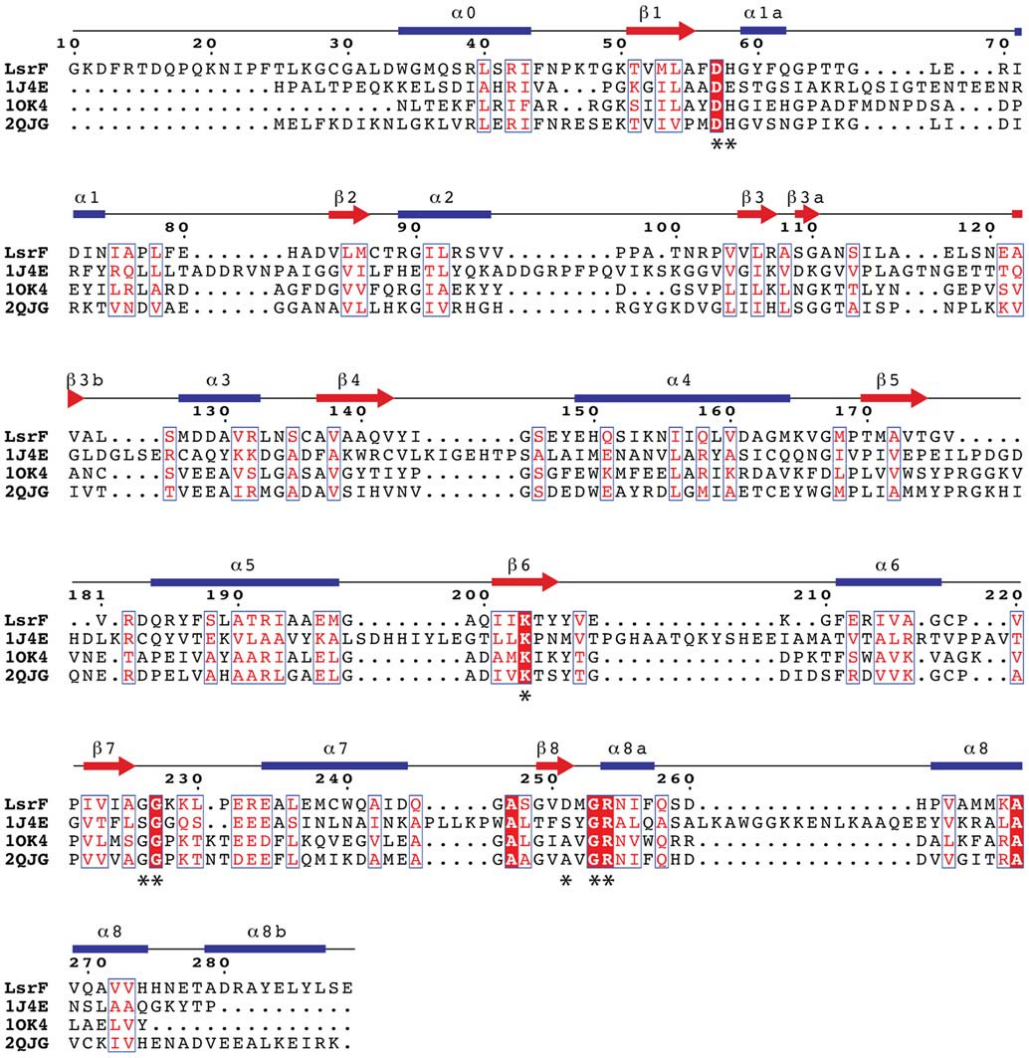
Structure-based sequence alignment highlights conservation of binding-site and potential catalytic residues. Structure-based alignments [47] were calculated for LsrF with rabbit FBPA (1J4E), *T. tenax* FBPA (1OK4), and *M. jannaschii* ADH synthase (2QJG). Identical residues are in white on red, conserved residues are in red (boxed). Secondary structure (from LsrF) is indicated above the sequence: blue bars are α-helices and red arrows are β-sheets. Residues implicated as either hydrogen bonding to the ligand phosphate or catalytic are indicated with an asterisk; these residues are disproportionately conserved. Numbering follows the LsrF sequence.

LsrF crystallizes as a decamer with each monomer having an (αβ)_8_-barrel fold (Fig. 2a), a ubiquitous fold commonly seen in proteins catalyzing aldolase reactions [28]. In a departure from the typical (αβ)_8_-barrel fold, the first β-strand of the LsrF barrel does not start until residue 51. Instead, the first 25 residues of the chain extend away from the barrel and pack against other subunits in a form of domain swapping previously unseen in aldolases (Figs. 2b and 3b). (There is no interpretable density for residues 1–9, but the orientation of adjacent residues make it impossible for these residues to pack against the (αβ)_8_-barrel of their own chain.) After a short coil, residues 34–43 (α0) form an α-helix that both caps the bottom of the barrel and makes extensive interactions with neighboring monomers (Figs. 2a, 2c, and 4). Following the first β-strand, the (αβ)_8_-barrel fold is briefly interrupted by a small stretch of α-helix (residues 59–62, α1a) that packs against a neighboring monomer. A relatively large loop joins β3 to α3 and is bounded by two short β-strands (residues 109–110, β3a, and 122–123, β3b) that anchor this loop. The canonical (αβ)_8_-barrel then continues until a final interruption when residues 254–257 (α8a) form an α-helix prior to α8. After the final helix of the (αβ)_8_-barrel, the C-terminal residues form an α-helix (α8b) that largely lies in the groove between the seventh and eighth helices of the barrel.

The LsrF oligmer has a disk-like structure, with two rings of five monomers stacked on top of each other giving rise to a decamer with D5 symmetry (Fig. 3). Each ring has a diameter of approximately 110 Å and has a central pore of about 15 Å diameter. Monomers make extensive contacts with the two adjacent subunits in the pentamer (subunit A interfaces with B and E, Fig. 3a). These contacts are largely hydrophobic, containing only two salt bridges (between residues Asp 128 and Arg 89 from chain A and Lys165 and Asp161 from chain B, respectively) and eleven potential hydrogen bonds. Most of the interactions between chains occur through the α-helices of the (αβ)_8_-barrels; in the A–B interface, helices 1a, 2, and 3, and the large loop between β3 and α3 from subunit A interact with helices 4, 5, and 6 from subunit B, burying some 1200 Å^2^ of surface area on each monomer. Since any given monomer participates in two of these interactions, pentameric interactions bury 18% of the solvent accessible surface area.

A second ring is related to the first by 2-fold symmetry axes perpendicular to the 5-fold axis of the pentamer. The stacked rings have a total height of approximately 70 Å, and the central pore runs this full length. Monomers stacked on top of each other (A and F; Fig. 3b) have extensive interactions that are significantly enhanced by a ‘swapping’ of N-terminal residues. In this swap, residues 10–24 extend away from the (αβ)_8_-barrel formed by their chain and pack into the interface between two adjacent monomers in the other pentamer, burying 460 Å^2^, or 21% of the accessible surface area of this swapped coil. The rest of the A-F interface is largely composed of contacts involving helix α0 and the loops after helices 2 and 3. In total, nearly 2100 Å^2^ are buried in this interface, 15% of the total accessible surface area.

One final interaction is due to the interface of the type seen between monomers B and F in Fig. 3b and is largely caused by the swapped 34 N-terminal residues. While this swapped coil packs chiefly against its direct neighbor from the other ring (i.e. A and F, Fig. 3b), it also makes contacts with α6 from the other monomer. In this case, 900 Å^2^ of surface area is buried, though the value may be even larger if residues 1–9 (disordered in the structure) also contribute to this interface. When all of the interfaces are considered, approximately 40% of the total surface area is buried in oligomer formation, suggesting that the decamer is likely the predominant form of LsrF in vivo. Consistent with this conclusion, only decamers were observed in gel filtration experiments (data not shown).

### Structure of the Ligand Binding Site

**Figure 5:**
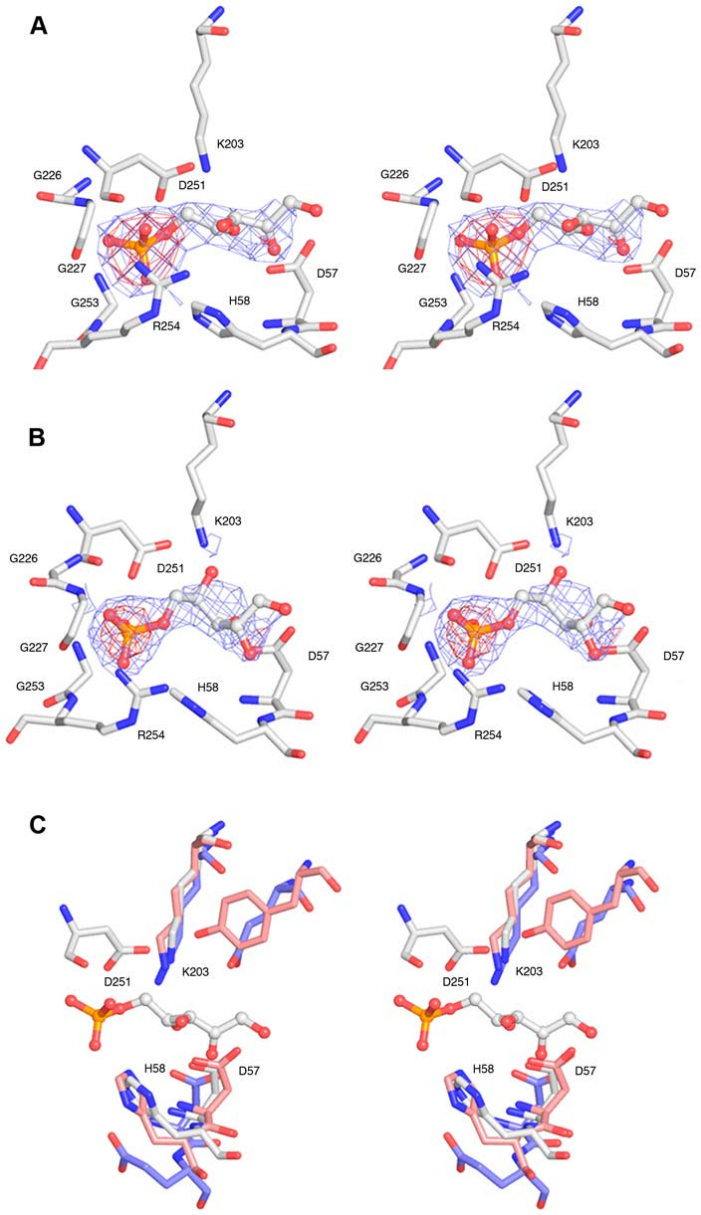
The LsrF ligand binding site and potential catalytic residues. A. Stereoview of ribulose-5-phosphate bound to LsrF showing 20-fold NCS averaged 2F_0_-F_C_ electron density. Density was contoured at 4.0 (red) and 2.0 (blue) σ and truncated 2.0 Å from ligand atoms. The position of the phosphate is unambiguous, and the general path of the ligand is clear. B. Stereoview of ribose-5-phosphate bound to LsrF showing 20-fold NCS averaged 2F_0_-F_C_ electron density. Density was contoured at 5.0 (red) and 2.0 (blue) σ and truncated 2.0 Å from the ligand. The position of the phosphate is unambiguous, and the general path of the ligand is clear. C. Structural alignment of key catalytic residues from rabbit (blue bonds; 1J4E) and *T. tenax* (red bonds; 1OK4) FBPA with LsrF (white bonds). Ribulose-5-phosphate from LsrF is shown in ball and stick form. Residue numbering follows LsrF.

To identify the catalytic site of LsrF, we determined the structure of the protein in complex with two P-AI-2 analogues: ribose-5-phosphate (R5P) and ribulose-5-phosphate (5RP) (Fig. 1). In both structures, the ligand electron density allowed definitive placement of the phosphate group and illustrated the general path of the carbon chain, but was of insufficient quality for unambiguous placement of all ligand atoms (Fig. 5a, b). As an independent confirmation of placement of the phosphate, the LigandFit module of PHENIX [34] was used as an automated means for placing the ligands. The automated procedure positioned the phosphates in the same location as was modeled manually.

R5P and 5RP bind LsrF in the same location, near the entrance to the (αβ)_8_-barrel with the phosphate group oriented towards the coils following β7 and β8 (Fig. 2a & c). The phosphate is located near the positively charged side chain of Arg254 and is positioned to form hydrogen bonds with the side chain of His58, the side chain and main chain of Arg254, and the main chain of three glycine residues: 226, 227, and 253 (in all cases with N or NH groups) (Fig. 5a, b). The ligand then extends across the center of the (αβ)_8_-barrel, away from strands 7 and 8, in a largely polar environment. In particular, Lys203 is adjacent to the ligand; this residue has potential significance for the mechanism, as equivalently positioned lysines are responsible for Schiff base formation in other aldolases (below). No large conformational changes were observed upon ligand binding, though there were small movements in a few binding site residues, most notably Asp251 and Met252 (with α-carbons shifting by 0.5–2.0 Å). A single water was built in the binding site of unliganded LsrF; this water is displaced upon ligand binding.

## Discussion

While AI-2 mediated quorum sensing has been identified in many bacterial species, the benefits bacteria gain from this communication are not fully understood. The presence of genes not involved in AI-2 transport in the *lsr* operon (*lsrF* and *lsrG*) raises questions about the eventual fate of internalized AI-2. Experiments studying regulation of the *lsr* operon in Δ*lsrF* mutants of *S. typhimurium* have implicated LsrF in P-AI-2 processing, though biochemical studies have also raised the possibility that LsrF acts on a product of the reaction involving P-AI-2 and LsrG or another P-AI-2 adduct [20], [21]. The structure of LsrF and complexes with P-AI-2 analogues presented here strongly suggest that LsrF is a class I aldolase.

### Fold Comparisons with other Aldolases

NCBI-Blast identifies LsrF as belonging to the TIM phosphate binding superfamily and, in searching for conserved domains, all e-values better than 1e-5 suggest it to be an aldolase (the strongest match, with an e-value of 5e-173, is with the aldolase cluster PRK08227). A DALI search using the LsrF monomer structure presented here identified two very similar structures (Z-scores greater than 25 and RMSD less than 2.0 Å), along with a large number of more distantly related structures that share the ubiquitous TIM barrel fold. The top DALI hits are the *M. jannaschii* ADH synthase, which catalyzes a transaldol reaction (PDB ID: 2QJG) [31], and the FBPA from *Thermoproteus tenax* (PDB ID: 1OJX) [30]. These proteins have low sequence identity with LsrF (31% and 25% respectively), but nonetheless share essentially identical folds, with the exception of the domain swapping involving the N-terminus of LsrF.

Domain swapping has been observed previously in the (αβ)_8_-barrel fold family, initially by Huang *et al* in phosphoenolpyruvate mutase where C-terminal residues are swapped, giving rise to dimers that further assemble into a tetramer [35]. C-terminal domain swapping has also been observed in the *E. coli* fructose-6-phosphate aldolase, FSA [36]. Like LsrF, FSA crystallizes as a decamer, but the domain swapping is C-terminal and occurs between subunits in the same pentamer, whereas in LsrF the swapping is N-terminal and occurs between subunits in different pentamers, presumably stabilizing the decameric form of the complex (Fig. 3b). Interestingly, no domain swapping is observed in the decameric structures of *T. tenax* FBPA or *M. jannaschii* ADH synthase, indicating that it is not a requirement for formation of a stable aldolase decamer.

### Comparison of the LsrF active site with other aldolases

The P-AI-2 analogues ribose-5-phosphate and ribulose-5-phosphate bind to LsrF in the same general position that other aldolases bind phosphorylated substrates (e.g. *E. coli* DERA and FBPA, *T. tenax* FBPA and *M. jannaschii* ADH synthase), suggesting that the canonical aldolase active site is conserved in LsrF. Structural-based sequence alignments with *T. tenax* FBP aldolase and *M. jannaschii* ADH synthase, the two most closely related aldolase structures, and *E. coli* FBP aldolase show that residues in the ligand binding site are more highly conserved than one would expect based on overall sequence identities (Figs. 4 and 5c), supporting the premise that these residues are important for LsrF substrate binding and activity. Notably, most of residues that hydrogen bond with the phosphate of the ligand in LsrF (Arg254 and Gly 226, 227, and 253) are structurally conserved in all four structures, and the remaining residue (His54) is conserved in *T. tenax* FBPA and *M. jannaschii* ADH synthase, but not *E. coli* FBPA, where it is replaced by a Glu. Examination of crystal structures of complexes of these proteins with phosphorylated ligands (1OK4, 2QJG, and 1J4E) shows that the conserved residues are positioned to form hydrogen bonds with the phosphoryl group of the ligand just as they are in LsrF.

The defining catalytic residue for a type I aldolase is a lysine that forms a Schiff base with the substrate. Structural alignments of LsrF with a variety of type I aldolases, including FBP aldolase from rabbit and *T. tenax*, ADH synthase from *M. jannaschii*, and transaldolase B, DERA, and 2-keto-3-deoxy-6-phosphogluconate (KDPG) from *E. coli* show structural conservation of the catalytic lysine with LsrF K203. Other catalytically significant residues vary across different aldolases, but nonetheless potential catalytic residues in LsrF can be identified from structural comparisons with FBP aldolase from rabbit and *T. tenax* and ADH synthase from *M. jannaschii*. These aldolases have an aspartate residue that acts as a general base, facilitating the carbon-carbon bond cleavage (or formation) by deprotonating an adjacent hydroxyl. The aspartic acid is then thought to donate the proton back during the reforming of the imine [29], [31], [37]. This asparate is structurally conserved in LsrF (Asp57; Fig. 5c) and is well positioned to participate in catalysis as a general acid/base.

The identity of the catalytic residue that participates in the dehydration of the carbinolamine during formation of the Schiff base differs in the various species. In most aldolases, including rabbit FBP aldolase, the residue is a glutamate adjacent to the catalytic lysine [29]. In *T. tenax* FBP aldolase, and *M. jannaschii* ADH synthase the catalytic glutamate is not conserved; instead, a tyrosine is positioned to act as a proton donor [30], [31]. Neither of these residues is structurally conserved in LsrF. Although a tyrosine (205) is adjacent to the position occupied by the catalytic Tyr in the other enzymes, it is too distant from the catalytic lysine (6.8 Å) to reasonably participate in catalysis. Rather, there is an aspartate (251) located only 2.9 Å from the K203, though on the other side of the lysine from the catalytic glutamate in rabbit FBP aldolase (Fig. 5c). This location, occupied by a serine in rabbit FBP aldoase and alanine in the other close homologues, makes Asp251 a very plausible replacement for the catalytic glutamate/tyrosine in other aldolases.

### Conclusion

The structures presented here strongly support the classification of LsrF as a class I aldolase, due to overall structural homology, the conservation of key catalytic residues, and conservation of the ligand binding site. Thus far, we have been unable to detect the products of the LsrF reaction in vitro, either by NMR or TLC using radiolabled substrate, in the presence or absence of the other P-AI-2 processing enzyme LsrG (results not shown). While previous work has implicated P-AI-2 [21] or an adduct of P-AI-2 [20] as the likely substrate for LsrF, it is possible that additional enzymatic processing of P-AI-2 or an additional co-factor is necessary for activity, and we are conducting genetic and biochemical experiments to address these possibilities.

If, as working model, we consider LsrF to act directly on P-AI-2 via an FBPA-like mechanism, we would expect the highly conserved catalytic K203 to form a Schiff base through nucleophilic attack on the carbonyl carbon one position away from the phosphate of the substrate (C4 of P-AI-2), leading to the breaking of the C2-C3 bond and the formation of acetate and dihydroxyacetone phosphate (DHAP). (It should be noted that hydration and keto-aldol isomerization would be necessary to make P-AI-2 an appropriate substrate for this reaction.) Intriguingly, prior work has shown that DHAP represses *lsr* transcription in an LsrR-dependant manner [19]. Thus, LsrF could function to reduce *lsr* transcription not only by reducing the amount of P-AI-2 present in the cell as previously suggested [21], but also by catalyzing the formation of an inhibitor of *lsr* transcription.

Further biochemical characterization of the LsrF reaction will be necessary to fully understand the role LsrF plays in AI-2 mediated quorum sensing, and the structures presented here provide details that will be of utility in the design of these experiments.

## Materials and Methods

### Overexpression and purification of LsrF

*E. coli* LsrF was cloned into plasmids pGEX-4T1 and pDEST-HisMBP for overexpression as glutathione*-S*-transferase and dual His_6_-maltose-binding-protein fusions, respectively. Plasmids were transformed into *E. coli* strain BL21, and cultures were grown in Luria broth (Sigma-Aldrich) at 37°C to an OD_595_ of 0.3. The temperature was then changed to 22°C, and, when the culture reached an OD_595_ of 0.9, protein expression was induced by the addition of 0.1 mM isopropyl β-D-thiogalactopyranoside. After induction, the bacteria were grown for 15 hours at 22°C before harvesting by centrifugation.

Cells producing the GST-LsrF fusion were resuspended in 25 mM Tris, pH 8.0, 150 mM sodium chloride, 5 mM DTT, 2.5 µg mL^−1^ DNase, and protease inhibitors (2.5 µg mL^−1^ aprotinin, 2.5 µg mL^−1^ leupeptin, 1 mM Pefablock (Roche)), while cells producing the His_6_-MBP-LsrF fusion were resuspended in 25 mM HEPES, pH 8.0, 200 mM sodium chloride, 25 mM imidazole, 1 mM β-mercaptoethanol, 2.5 µg mL^−1^ DNase, and protease inhibitors (2.5 µg mL^−1^ aprotinin, 2.5 µg mL^−1^ leupeptin, 1 mM Pefablock). In both cases, the cells were lysed using a M-110Y Microﬂuidizer (Microfluidics) and the lysates clarified by centrifugation.

The GST-LsrF fusion was purified by affinity chromatography using glutathione agarose (Sigma-Aldrich). The fusion protein was digested with thrombin for 12 hours at 4°C while still bound to the glutathione agarose. LsrF was eluted from the agarose column in 25 mM Tris, pH 8.0, 150 mM sodium chloride, and 1 mM DTT. The resulting protein solution was diluted with 25 mM Tris, pH 8.0, 1 mM DTT, to a NaCl concentration of 75 mM. LsrF was then further purified by ion exchange chromatography using a SourceQ column (GE Healthcare) with a gradient from 0 to 1 M NaCl. As a final purification step, the protein was subjected to size exclusion chromatography on a Superdex 200 column (GE Healthcare), eluting in 25 mM Tris pH 8.0, 1 mM DTT, and 150 mM NaCl. The protein was concentrated to 9.6 mg ml^−1^ for crystallization.

The His_6_-MBP-LsrF fusion was also purified by affinity chromatography, but in this case using NiNTA agarose (QIAGEN). The fusion protein was eluted from the column using a gradient from the resuspension conditions to 25 mM HEPES pH 8.0, 200 mM sodium chloride, 250 mM imidazole as described in Tropea *et al* [38]. Protein containing fractions were pooled, and the concentration of imidazole was reduced to 25 mM by diluting with 25 mM HEPES, pH 8.0, 200 mM NaCl. The His_6_-MBP tag was then digested from the LsrF using His_6_-TEV protease [38]. The tag and protease were removed by passing the solution over NiNTA resin, and the resulting LsrF solution was diluted to 50 mM NaCl with a 25 mM HEPES, pH 8.0. The LsrF was purified by ion exchange and size exclusion chromatography as described above, and the resulting LsrF was concentrated to 8.2 mg ml^−1^.

### Crystallization and Structure Determination

Crystals of LsrF were grown via the hanging drop method with a well solution of 22% PEG 400, 200 mM MgCl_2_, 100 mM Tris pH 8.0. Unliganded crystals were grown from the pGEX-4T1 derived protein while protein for the ligand-soaked crystals came from the pDEST-MBP construct.

Unliganded crystals were soaked in 100 mM Tris pH 8.0, 25 mM MgCl_2_, 27.5% PEG 400 for one minute and flash frozen in the diffractometer's cryostream. Data were collected at 100K using an R-AXIS-IV image plate detector mounted on a Rigaku 200HB generator. The crystals (P_1_, a = 78.50 Å, b = 104.61 Å, c = 171.67 Å, α = 89.88°, β = 79.31°, γ = 89.61°) diffracted to 2.9 Å resolution. Ligand was introduced to LsrF crystals by soaking the crystals in 100 mM Tris pH 8.0, 100 mM MgCl_2_, 27.5% PEG 400, 100 mM ligand (either ribulose-5-phosphate or ribose-5-phosphate, Sigma-Aldrich) for five minutes. Crystals were flash frozen in liquid nitrogen and data were collected at 100K at NSLS beamline X26C. The ribose-5-phosphate crystal (P_1_, a = 78.35 Å, b = 105.45 Å, c = 173.42, α = 89.51°, β = 79.79°, γ = 90.34°) diffracted to 2.5 Å resolution while the ribulose-5-phosphate crystal (P_1_, a = 78.741 Å, b = 107.10 Å, c = 169.52, α = 90.00°, β = 102.62°, γ = 90.00°) diffracted to 2.9 Å resolution. Data were processed using Denzo, Scalepack [39], and CCP4 [40]. It should be noted that while the unit cell of the ribulose-5-phosphate crystals is, in appearance, potentially monoclinic, the data does not scale well as monoclinic at higher resolutions. Moreover, the apparent large variation in the β angle for the ribulose-5-phosphate crystal is due to an alternative convention selected by Denzo rather than a significantly different cell.

The structure of unliganded LsrF was determined via molecular replacement with PHENIX [34], using ADH synthase from *M. jannaschii* (PDB ID: 2QJG, 31% sequence identity) as the search model. A 20-fold NCS averaged map was calculated and the model built using Coot [41]. Because of the high degree of NCS, reflections were selected for the R-free set in thin resolution shells using DATAMAN [42]. The structure was refined to 2.9 Å using PHENIX and REFMAC [43], using NCS constraints. The model contains 2 copies of the LsrF decamer, though weak density made it impossible to model the N-terminal 9 residues, the C-terminal 2 residues, and residues 177–180, an apparent surface loop. The model exhibits good geometry (Table 1), with only eleven of 5440 residues outside the allowed region of the Ramachandran plot (calculated by Coot). The final model also includes 241 water molecules, and has a final R_cryst_ of 0.209 and R_free_ of 0.229.

**Table 1:** Crystallographic data and refinement statistics.

Data (highest resolution shell in parenthesis)	Unliganded	Ribose-5-phosphate	Ribulose-5-phosphate
Resolution (Å)	2.9 (2.900–2.975)	2.5 (2.500–2.565)	2.9 (2.900–2.975)
Unique reflections	113374 (7444)	121116 (5138)	107205 (7028)
R_merge_	0.092 (0.361)	0.086 (0.380)	0.090 (0.436)
Mean I/σI	9.1 (1.9)	7.5 (1.8)	8.7 (2.0)
Completeness (%)	95.3 (90.5)	81.7 (38.6)	94.2 (83.7)
Multiplicity	1.8 (1.7)	2.1 (1.7)	1.7 (1.6)
**Refinement**			
R_cryst_/R_free_	0.209/0.229	0.205/0.235	0.195/0.228
RMSD bond length (Å)	0.013	0.014	0.013
RMSD bond angle (°)	1.317	1.358	1.178
Number of atoms per ASU	42541	42874	42916
Average B factor (Å^2^)			
Protein	37.04	35.77	25.42
Water	23.87	25.07	16.89
Ligands		80.09	54.18
Ramachandran Plot			
Most favored (%)	97.1	96.0	93.8
Allowed (%)	2.7	4.0	6.2
Disallowed (%)	0.2	0.0	0.0

The liganded structures were determined by molecular replacement via PHENIX, though in these cases the unliganded LsrF structure was used as the molecular replacement model and reflections for the R_free_ set were selected randomly rather than in resolution shells. Refinement parameters for the ligands were calculated using the eLBOW module of PHENIX. The ribose-5-phosphate structure was refined via PHENIX and REFMAC, using NCS constraints, to 2.5 Å resolution, with R_cryst_ = 0.205 and R_free_ = 0.235. The size of the unit cell made it difficult to collect a complete data set at high resolution, and only 39% of the possible reflections were measured in the highest resolution shell. However, the lack of completeness is offset by the high degree of NCS (20-fold), making it reasonable to include data to this resolution. The final model included one ribose-5-phosphate per chain and 334 water molecules. The ribulose-5-phosphate structure was refined to 2.9 Å resolution via PHENIX and REFMAC, with NCS constraints, and the final model (R_cryst_ = 0.195, R_free_ = 0.228) includes one ribulose-5-phosphate per chain and 376 water molecules. Both models are missing the same residues as the unliganded model (1–9, 177–180, and 290–291) and have good geometry (Table 1), with either one (LsrF/ribulose-5-phosphate) or zero (LsrF/ribose-5-phosphate) of 5440 residues outside allowed regions of the Ramachandran plot. The position of the electron-rich phosphate is clear for both ligands, but the density of the carbon backbone was relatively poor, revealing the general path of the ligand but not specific details and leading to high B-factors.

Coordinates and structure factors for unliganded LsrF were deposited in the PDB with accession number 3GKF. Coordinates and structure factors for liganded LsrF were deposited in the PDB with accession numbers 3GLC (ribose-5-phosphate) and 3GND (ribulose-5-phosphate).

The secondary structure elements were determined using DSSP [44] and analysis of subunit interfaces used PISA [45]. All molecular images were generated using PyMOL [46]. Structural alignments were calculated with MAMMOTH-mult [47] and the alignment figure was produced with ESPript [48].
